# Top–Down Modulation on the Perception and Categorization of Identical Pitch Contours in Speech and Music

**DOI:** 10.3389/fpsyg.2016.00817

**Published:** 2016-06-02

**Authors:** Joey L. Weidema, M. P. Roncaglia-Denissen, Henkjan Honing

**Affiliations:** Music Cognition Group, Amsterdam Brain and Cognition, Institute for Logic, Language, and Computation, University of AmsterdamAmsterdam, Netherlands

**Keywords:** pitch perception, language, music, categorical perception, tone languages

## Abstract

Whether pitch in language and music is governed by domain-specific or domain-general cognitive mechanisms is contentiously debated. The aim of the present study was to investigate whether mechanisms governing pitch contour perception operate differently when pitch information is interpreted as either speech or music. By modulating listening mode, this study aspired to demonstrate that pitch contour perception relies on domain-specific cognitive mechanisms, which are regulated by top–down influences from language and music. Three groups of participants (Mandarin speakers, Dutch speaking non-musicians, and Dutch musicians) were exposed to identical pitch contours, and tested on their ability to identify these contours in a language and musical context. Stimuli consisted of disyllabic words spoken in Mandarin, and melodic tonal analogs, embedded in a linguistic and melodic carrier phrase, respectively. Participants classified identical pitch contours as significantly different depending on listening mode. Top–down influences from language appeared to alter the perception of pitch contour in speakers of Mandarin. This was not the case for non-musician speakers of Dutch. Moreover, this effect was lacking in Dutch speaking musicians. The classification patterns of pitch contours in language and music seem to suggest that domain-specific categorization is modulated by top–down influences from language and music.

## Introduction

Both speech and music perception focus on the acoustic signal, which is organized in a temporally discrete and hierarchical manner (McMullen and Saffran, 2004; Patel, 2008). Pitch is a fundamental and highly perceptual acoustic attribute of both language and music. In language, pitch is generally continuous and curvilinear, in music often relatively discrete (e.g., Zatorre and Baum, 2012). A question of particular interest concerns whether pitch in both domains is governed by domain-specific cognitive mechanisms (Peretz and Coltheart, 2003; Peretz, 2009) or whether it is processed by domain-general, shared processing mechanisms that span over both domains (Patel, 2008, 2012; Asaridou and McQueen, 2013). Making a direct comparison between domains is challenging when considering the acoustic similarities, as well as the structural and functional differences between pitch in speech and pitch in music (see, e.g., Patel, 2012; Peretz et al., 2015). Whether or not mechanisms governing pitch processing are shared or distinct, understanding how these mechanisms operate in each domain is of great relevance to both language and music cognition. The present study will address this premise by investigating domain-specific perception of pitch in both language and music using identical pitch contours embedded in linguistic or musical contexts.

Pitch processing in speech and music can be categorized in two distinct classes: interval relations, and contour processing. Pitch relations based on interval are computed by comparing the relative distance between successive sounds, while contour is processed in relative directional terms of ups and downs (Dowling and Fujitani, 1971; Dowling, 1978). Interval processing is considered a domain-specific, musical skill (e.g., Zatorre and Baum, 2012). Contour processing is prevalent in both domains, with a possible biological basis (e.g., Nazzi et al., 1998; Thiessen et al., 2005). It is considered an essential component of *musicality* (Honing et al., 2015), fundamental to both language and music processing.

Tracking the contour of a fundamental frequency (F0) in both speech and music shows a number of similarities with regard to pitch height (*frequency*) and direction (*contour*; e.g., Gandour and Harshman, 1978; Chandrasekaran et al., 2007). In music, contour processing concerns the directional relationships between tones that make a melody (Dowling and Fujitani, 1971). In language, non-tonal specifically, contour processing refers to a fundamental aspect of prosody that, amongst intensity and durational factors, forms the intonational constituents of language (Cruttenden, 1997). It guides a listener in distinguishing questions from statements, and enables the detection of emotive states in utterances for example (e.g., Bänziger and Scherer, 2005).

Along side intensity and duration markers, a primary cue for tone languages, specifically, is the use of lexically contrastive pitch contours on syllables. These contours are characterized by height and direction of the F0 to differentiate between different word meanings (Gandour, 1978). Mandarin, for example, makes use of four distinct pitch contours that are lexically contrastive: high level, rising, dipping, and falling (Yip, 2002). Because of the similar acoustic properties of lexical and musical pitch in the frequency dimension, tone language speakers and musicians are often compared to investigate the relationships between speech and music processing.

Absolute pitch (AP) refers to the ability to name a musical note without a given reference, and its genesis has been related to both genetic and environmental factors (Baharloo et al., 1998; Gregersen, 1999; Gregersen et al., 2001; Theusch et al., 2009). In speakers of tonal languages there tends to be a higher prevalence of AP, particularly in the ability to associate a specific verbal label with a specific pitch (e.g., Deutsch et al., 2004). AP also seems to correlate with the age of onset for musical training in both tonal and non-tonal speakers (Deutsch et al., 2006, 2009): musically trained tonal speakers, however, demonstrate a greater prevalence. The above evidence suggests that while the genesis of AP contains a possible genetic component, the importance of environmental influences such as language and music appear significant.

Speakers of tone languages tend to demonstrate more effective perceptual performance for musical pitch than non-tonal speakers at both the behavioral and cortical level (Pfordresher and Brown, 2009; Bidelman et al., 2011a, 2013; Giuliano et al., 2011). Interestingly, sensory enhancement for pitch appears to operate bi-directional in that musicians tend to display more fine-grained (lexical) pitch perception in language (e.g., Delogu et al., 2010), and process linguistic pitch patterns more robust in both cortical and sub-cortical areas than non-musicians do (Wong et al., 2007; Chandrasekaran et al., 2009; Bidelman et al., 2011a,b; Hutka et al., 2015). This suggest that high-level training, such as learning an instrument, or long-term exposure to a tone language, can influence both top–down and bottom–up sensory encoding mechanism of pitch, and affect perception in both domains (see Krishnan and Gandour, 2014 for a review).

Categorical perception is an illustrative example of how cognitive representations exert top–down influence, and modulate perceptual mechanisms in domains such as language and music. It concerns a curious phenomenon in which the categories possessed and imposed by an observer tend to distort the observer’s perception. As such, we tend to classify the world in sharp categories along a single continuum, where change is often perceived not as gradual but in discrete classifications (see, e.g., Goldstone and Hendrickson, 2009 for a review). Categorical perception thus provides an excellent avenue for the investigation of the domain-specificity of pitch in language and music, as both domains tend to revolve around specific and highly learned categories.

Language experience, for example, has been shown to affect categorical perception of segmental features of phoneme perception in both adults and in infants (e.g., Liberman et al., 1957; Kuhl, 1991). Wang (1976) demonstrated that one’s language background affects the degree of categorical perception for pitch contours. Mandarin speakers exposed to gradations of native lexical tones demonstrated clear categorical boundary effects, which showed influence of native linguistic pitch categories. Non-tonal speakers, on the other hand, labeled categories more on clear psychophysical properties of the sound (e.g., the difference in frequency). Further studies investigating categorical perception of pitch contours have shown clear categorization effects modulated by language for both speech and non-speech in speakers of tonal and non-tonal languages (Pisoni, 1977; Hallé et al., 2004; Xu et al., 2006; Peng et al., 2010), and tonal speaking musicians (Wu et al., 2015).

In a multi-store model of categorical perception, Xu et al. (2006) posit how top–down interference effects from domains such as language can exert significant modulation on the way a pitch contour is categorized. In this model, sensory short-term memory contains fine-grained sensory codes that analyze pitch height, direction, and time before the memory trace moves on to a temporary buffer of short-term categorical memory. The memory trace moves on to long-term categorical memory before decisions are made regarding its categorical and domain-specific labels. This long-term categorical memory thus operates on bottom–up matching of the stimulus, but similarly creates top–down expectation that modulate perception.

Similar top–down categorical effects have been found in music perception, where musicians show clearly defined categories based on learned musical intervals (Siegel and Siegel, 1977; Burns and Ward, 1978; Zatorre, 1983; Burns and Campbell, 1994). Burns and Ward, for example, demonstrated that categorical perception is operant in the perception of musical intervals. Musically trained individuals were exposed to in and out of tune musical intervals and consequently demonstrated sharp categorical boundaries between major and minor. Remarkably, Burns and Campbell (1994) found boundary effects for categorical prototypes to be operant in both possessors of relative, and of AP. Much in the same way as linguistic categories are represented given a persons’ native language, this suggests that pitch information is encoded to discrete pitch categories of a learned musical scale which affect perception (see Thompson, 2013).

Previous work (e.g., Xu et al., 2006; Peng et al., 2010; Wu et al., 2015), demonstrated how top–down effects from language influences the identification of pitch contours in isolated speech and non-speech sounds at the word level. This study will use a similar paradigm but additionally will modulate listening modes between language and music perception in phrasal context. While previous studies have focused on the effect of musical expertise on pitch contour processing using both linguistic and musical materials in speakers of tone and non-tone languages (Schön et al., 2004; Magne et al., 2006; Marques et al., 2007; Mok and Zuo, 2012), to our knowledge, this is the first study that uses identical pitch contours embedded in clearly demarcated linguistic and melodic phrases. By assessing categorical identification in both domains independently, it will become possible to elucidate how mechanisms governing pitch contour processing operate differently in speech and music, respectively, depending on prior musical or linguistic experience.

The aim of the current study is to contribute to the question of domain-specificity of contour perception in a speech and musical listening mode by examining the categorical perception of pitch contours in three groups of participants: Mandarin native speakers, non-musician Dutch speakers, and Dutch musicians. Two of our subject groups represent pitch experts at different points of a continuum: from experts with pitch in speech to experts in music, with non-musician native speakers of Dutch as a control group.

**FIGURE 1:**
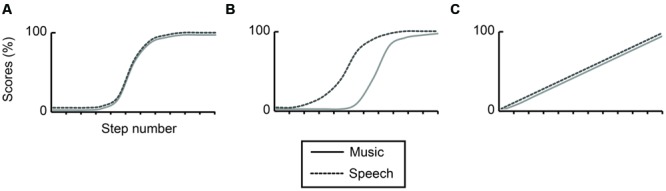
**Hypothesized levels of categorization: identical identification curves **(A)**, demarcated identification curves **(B)**, and absence of categorization **(C)** for a pitch continuum comparing speech (dotted line) and music (solid line).** The y-axis displays scores in percentages, the x-axis individual steps in the identification curve.

If contour perception operates identically in both domains, categorical identification will consist of similar, and overlapping sigmoid curves (**Figure 1A**). Alternatively, if contour perception operates differently in both domains, categorical identification will consist of differentiating sigmoid curves (**Figure 1B**). Otherwise, contour perception could be linear, continuous, and thus non-categorical, with no clear cut-off point in categorical identification for either domain (**Figure 1C**).

It is hypothesized that speakers of Mandarin will show clearly demarcated identification curves, and will identify pitch contours better in speech than in music as a result of experience in their native language (**Figure 1B**); that non-musician speakers of Dutch will show clearly demarcated categorical identification, and will perform equal across both conditions but worse than speakers of Mandarin and musicians (**Figure 1A**); and that musicians will identify contours identically across conditions (**Figures 1A,C**) as a function of expertise and acuity in acoustic processing (e.g., cf. Patel, 2011; Hutka et al., 2015).

## Materials and Methods

### Participants

Forty-eight participants (21 males) took part in the experiment: 32 were university students, and 16 participants were professional or highly trained musicians. All participants reported normal hearing and eyesight, and no known history of neurological disorders. Participants provided formal written consent before the start of the experiment, and were all paid a fee for their participation. The ethics committee of the Faculty of Humanities of the University of Amsterdam approved the study.

Sixteen speakers of Mandarin (nine males: mean age: 26, *SD* = 3.74) formed the tonal language group. All spoke Mandarin as their first language, and reported speaking their native language on a daily basis. Participants in this group had little musical training (*M* = 3.38 years, *SD* = 2.77), and none had received any form of instruction in the previous 5 years. 16 non-musician speakers of Dutch formed the non-tonal language non-musician group (three males: mean age: 23.25, *SD* = 2.52). They had received little musical training (*M* = 2.63 years, *SD* = 2.68), and none had received formal instruction in the previous 5 years. Mandarin and non-musician Dutch speakers did not differ statistically from each other with regard to years of musical training (*p* = 0.72). 16 highly trained musicians formed the musician group (nine males: mean age: 26.31, *SD* = 6.38). Participants in the musician group were all amateur or professional musicians (years of training *M* = 18.88 years, *SD* = 5.97). The primary instruments played by the participants were: bassoon (1); clarinet (2); flute (3); harpsichord (1); piano (5); saxophone (1); viola (2); and violin (1).

### Stimuli

Two sets of stimuli were created: speech items, and musical counterparts. Experimental items for the speech condition consisted of three disyllabic minimal pairs in Mandarin: i.e., rising (tone 2) with falling (tone 4) counterparts. These minimal pairs (e.g., tian1 ming2 (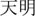: ‘*dawn*’) vs. tian1 ming4 (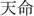: ‘*destiny*’)) differed only with regard to meaning: i.e., the pitch contour of the last syllable (*falling* vs. *rising*). Items were matched in terms of lexical frequency (Cai and Brysbaert, 2010) to control for possible frequency effects: all *p*s > 0.5). Words were read out loud by a female native speaker of Mandarin at a constant rate in a sound attenuated booth, and recorded at a sampling rate of 44.1 kHz.

**FIGURE 2:**
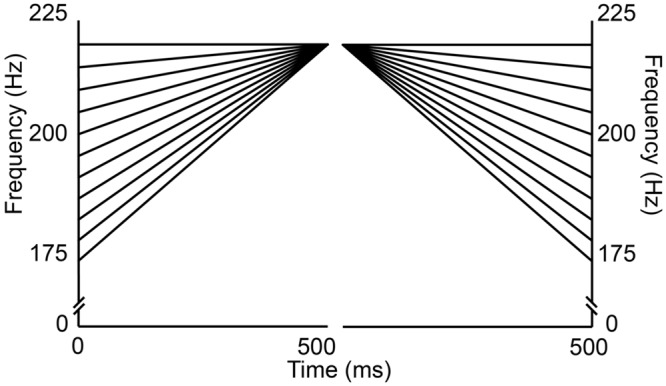
**Frequency (y-axis) and time (x-axis) chart for the pitch continua.** The left side depicts the 11 individual steps for the rising tone; the right side each step for the falling tone.

A tonal continuum was created by dividing a finite pitch space into 11 equal sized steps using equivalent rectangular bandwidth (ERB) psychoacoustic scaling (see Xu et al., 2006). This scale was chosen as it more closely approaches the frequency sensitivity along the basilar membrane (Greenwood, 1961; Hermes and van Gestel, 1991). Frequency contours were modeled by a linear function from *rising* to *flat*, and *flat* to *falling* (see **Figure 2**). The largest distance was 45 Hz (≈4 semitone) and the smallest 4.25 Hz (≈0.3 semitone). Onset frequency for step 1 was set to 175 Hz, and offset frequencies of each of the 11 items were 220 Hz. All steps were separated by an equal size of 0.09 ERB (see **Table 1**).

**Table 1:** Onset frequency and step size of the F0 in Hz and ERB scaling.

Step number	*f* (Hz)	*E* (ERB)	Step size (Hz)	Step size (ERB)
1	220.00	6.135	4.76	0.09
2	215.24	6.045	4.70	0.09
3	210.55	5.955	4.64	0.09
4	205.91	5.865	4.58	0.09
5	201.33	5.775	4.53	0.09
6	196.80	5.685	4.47	0.09
7	192.33	5.595	4.41	0.09
8	187.92	5.505	4.36	0.09
9	183.56	5.415	4.31	0.09
10	179.25	5.325	4.25	0.09
11	175.00	5.235		

The modeled linear frequency contours were used to replace the original contours of the last syllable of the disyllabic speech items, while the first syllable was kept unaltered (i.e., curvilinear). Using PSOLA to manipulate the stimuli in both the time and frequency domain (Moulines and Charpentier, 1990) in PRAAT software (Boersma and Weenink, 2014), speech items were resynthesized with each of the 11 pitch contours. The procedure for replacing the original F0-contour was identical for both falling and rising tones.

**FIGURE 3:**
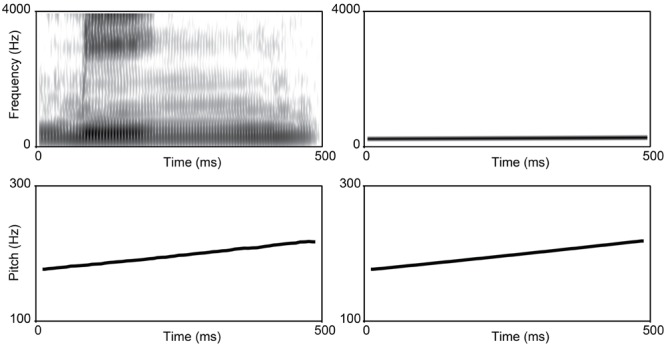
**Comparison of speech **(left)** vs. music **(right)** stimuli.** The top left box displays a broadband spectrogram of step 1 from the rising continuum for speech; the top right box displays a narrowband spectrogram of step 1 for music. The bottom panels display the corresponding pitch contours of the F0 for speech **(left)** and music **(right)**. All frequencies are displayed in Hz.

To create melodic counterparts to the speech items, the pitch contours from the tonal continua were extracted using a high-resolution pitch extraction algorithm in PRAAT (Boersma and Weenink, 2014). These pitch points were used to resynthesize homologous sinusoidal gliding tones. For both sets of stimuli (speech and melody), syllable and tone duration were normalized to 500 ms. These sinusoidal waveforms were identical with regard to pitch contour, amplitude and duration of the last syllable of the speech items without containing any of the phonetic content, or the natural harmonics found in language. The main acoustic difference between speech and musical stimuli was thus expressed in terms of spectral content (e.g., formant composition; see **Figure 3**).

In order to prime a speech listening mode, critical items were presented in a semantically neutral carrier sentence [*xia*^4^ *yi*^1^ *ge*^4^ *zi*^4^ *shi*^4^ (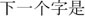): ‘*The next word is*’]. To ensure a musical listening mode, homologous pitch contours were placed after a melody comprised of discrete sinusoidal tones (C4, E4, C5, G4). With regard to temporal and metrical alignment, the melody was closely matched to the linguistic carrier. The total duration of both carriers was identical (1,500 ms). To facilitate pitch perception, a 500 ms inter-stimulus interval was placed before the presentation of the critical items. The total length of a single trial was thus 3,000 ms. Intensity profile was kept unaltered for speech, and constant for music. To ensure a comparable intensity of loudness between the two conditions, all stimuli were normalized to 80 dB.

### Procedure

All participants were tested individually in a sound attenuated booth. Participants listened to speech and musical stimuli in two separate conditions. Each condition was counterbalanced across participants. In both the speech- and the music-condition, there were six occurrences of each of the 11 pitch contours (each member of the minimal pair preceded by a flat, rising, and falling contour: 132 trials per condition) embedded in the final position of linguistic, and melodic phrases. Stimuli were pseudo-randomized with the sole restriction that no contour could be followed by an identical contour. Each trial was announced 500 ms pre-stimulus onset by a visual prompt (a white asterisk) on a black screen. There was a 1,000 ms interval between each trial: once a response had been entered, or after 2,500 ms had elapsed, the next trial would start. Stimuli were presented over two speakers at a consistent sound level.

Participants were carefully instructed to decide whether the last pitch contour they heard was rising, falling or flat by pressing the corresponding button on a keyboard as fast and accurately as possible in a three-alternative forced choice task (3 AFC: *rising, falling* or *flat*). Prior to the start of the experiment, a practice session consisting of 10 trials (four falling, four rising, and two flat tones) was performed, after which they received feedback. Accuracy rates were collected.

At the end of the experiment, musical background information was assessed by means of a written self-reported questionnaire on participants’ formal music training: number of years actively playing an instrument; numbers of hours daily practice; and numbers of instruments played. The entire procedure lasted approximately 60 min.

### Data Analysis

Perceptual sensitivity for each group, stimulus type and continuum, was assessed using d-prime (*d*’) scores. d-Prime values were computed by using the transformed *z-*scores that corresponded to the hit (*H*) and false alarm (*FA*) rates [i.e., *d*’ = *z*(*H*) – *z*(*FA*); Stanislaw and Todorov, 1999; Macmillan and Creelman, 2005]. Mean *d’* values were input to analysis of variance (ANOVA) using group (*Mandarin speakers, Dutch non-musicians and Dutch musicians*) as a between-subject factor, with continuum (*rising* vs. *falling*) and stimulus type (*speech* vs. *music*) as within-subject factors. Flat tones served as fillers and were disregarded from the analysis. Violations of sphericity were adjusted with Greenhouse–Geiser corrections, and pairwise comparisons with Bonferroni corrections were conducted where appropriate. Partial eta-squared ($\eta_{p}^{2}$) is reported as an estimate of effect size. To investigate categorical boundary position between speech and music, we conducted within-group Wilcoxon signed-rank tests pairwise comparisons for stimulus type and step number with mean accuracy rates for each step of the rising and falling continua separately.

## Results

**FIGURE 4:**
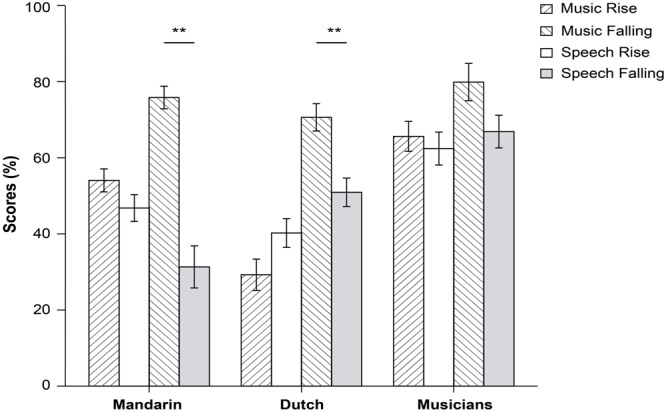
**Average scores per group in percentages, comparing continuum and stimulus type (^∗∗^*p <* 0.001)**.

**Table 2** contains the mean *d*-prime values, and **Figure 4** shows mean accuracy rates. Analyses revealed a significant three-way interaction between group, stimulus type, and continuum [*F*(2,45) 6.24, *p* = 0.004, $\eta_{p}^{2}$ = 0.22]; indicating that there were significant differences in the way the three groups behaved in identifying rising and falling tones in either music or in language. As a result, we conducted separate ANOVAs per group.

**Table 2:** Mean d′-prime measures (*d*′) and standard deviations for each group (Mandarin; Dutch non-musician; Dutch musician); and condition (music; speech).

	Music		Speech	
	Rising	Falling	Rising	Falling
Mandarin	2.13 (0.17)	2.68 (0.19)	1.84 (0.16)	1.25 (0.25)
Non-musician	0.70 (0.19)	1.13 (0.27)	0.64 (0.17)	0.89 (0.24)
Musician	2.07 (0.27)	2.62 (0.32)	1.76 (0.25)	1.98 (0.27)

For Mandarin speakers, there was a significant interaction between stimulus type, and continuum [*F*(1,15) 19.15, *p* = 0.001, $\eta_{p}^{2}$ = 0.56]. In music, falling tones were identified more accurately, and with a higher degree of sensitivity, than rising tones. In language the opposite pattern was found: falling tones were identified less accurate than rising tones. Furthermore, analysis revealed a main effect of stimulus type [*F*(1,15) 20.33, *p* < 0.0001, $\eta_{p}^{2}$ = 0.58]. Both accuracy rates and *d*-prime were significantly higher in music than in language.

For Dutch speaking non-musicians, we found a main effect of continuum [(*F*(1,15) 12.76, *p* = 0.003, $\eta_{p}^{2}$ = 0.46] but no significant interactions with stimulus type. In both music and language, falling tones were identified with higher accuracy and greater sensitivity than rising tones.

For musicians a main effect of stimulus type [*F*(1,15) 12.16, *p* = 0.003, $\eta_{p}^{2}$ = 0.45], and a main effect of continuum [*F*(1,15) 11.99, *p* = 0.003, $\eta_{p}^{2}$ = 0.44]. Musicians performed better on musical stimuli, and demonstrated a higher sensitivity to falling contours. The interactions between both variables was not significant [*F*(1,15) 3.44, *p* = 0.083].

**FIGURE 5:**
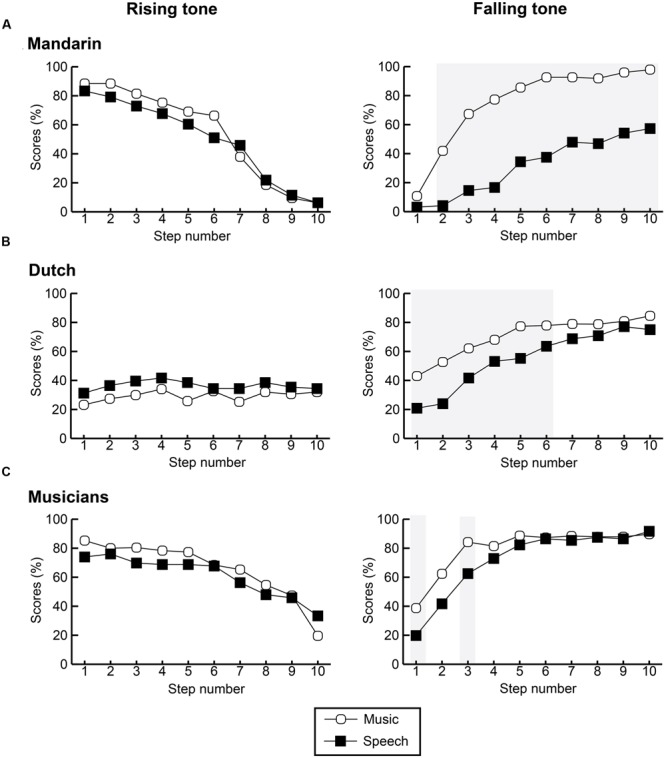
**Identification curves per step in each pitch continuum comparing between participant group and conditions for Mandarin participants **(A)**, Dutch non-musician participants **(B)**, and Dutch musicians **(C)**.** Left side illustrates rising tones in speech and music; right side contains values for falling tones. Gray shaded areas indicate significant differences between contexts per step.

Based on visual inspection of plotted performance, we conducted a within-group comparison of step-wise categorical identification in language vs. music for each tone separately to investigate whether categorical boundary position differed across speech and music. While none of the participant groups demonstrated significant differences for rising tones (all *p*s > 0.05), falling tones were categorized significantly different between both domains in all three groups (see **Figure 5** below).

Speakers of Mandarin demonstrated significant differences between categorization of the falling tone in all steps with the exception of step 1 (all *p*s < 0.001). While the difference for the first step is non-significant, accuracy is higher in music than in language [*M* = 11% (*SD* = 19) vs. *M* = 3% (*SD* = 7)]. Interestingly, the identification curve in language is far more sigmoid, hence categorical, than the shallower curve for music, and comparable to the sigmoid curves found for rising tones in both domains.

For Dutch non-musicians, the only significant differences occurred at steps 1–6 (all *p*s < 0.05) of the falling tone. The most significant accuracy differences were found at the first half of the continuum with higher accuracy rates for music than language. While the identification curves were still rather shallow, there was a larger tendency toward categorization of this contour when compared to the almost planate curve found for the rising tone in speech and music.

For musicians the only significant difference occurred at step 1 and step 3 (all *p*s < 0.05). It is interesting to note that for steps 1–5 musicians demonstrate higher accuracy rates for music than language, but that after this step differences between domains becomes negligible (all *p*s > 0.25). Compared between groups, the Dutch musician group demonstrated the most sigmoid shaped curve, hence categorization, in both domains.

## Discussion

The current study has investigated the identification of identical pitch contours in speech and in music. Speakers of Mandarin, Dutch non-musicians and Dutch musicians were presented with pitch contours in sentences and melodies and were required to indicate the direction of the last pitch contour (rising vs. falling) they heard. Results suggest that domain-specific perceptual mechanisms were employed differently by each participant group to identify pitch contours. Overall, participants performed better in music than in speech. A general tendency was observed for all groups to demonstrate a higher degree of categorization in response to falling tones – regardless of domain. The results, however, also show clear distinctions between the three groups in terms of accuracy and categorization.

Speakers of Mandarin displayed higher accuracy rates in music than speech, and clearly demarcated categorization in both domains. This group tended to demonstrate the most significant differences in terms of categorization between domains and pitch continuum. Against predictions, speakers of Mandarin demonstrated significant better performance in music than they did in speech – specifically for the falling tone. Such a direction-specific interference effect could suggest that an underlying canonical representation of a lexical tone interferes with contour perception. Thus, top–down mechanisms from language might have influenced the manner in which this specific pitch contour was categorized.

Perhaps the underlying representation of the falling tone in Standard Mandarin, which consists of a shorter falling ramp and greater intensity than the manipulated tones used in the current experiment, interfered with pitch perception in Mandarin speakers. It could also offer an explanation for the difference in categorical identification for this tone in speech and music. The finding that language-specific pitch categories can distort perception in a top–down manner for speakers of tonal languages corroborates findings from other studies (e.g., Bent et al., 2006; Peretz et al., 2011). The interference effect in the current experiment appeared to operate language-specific despite the low accuracy rates for this tone in music in the first steps of categorization. Although, some modulation from language might have interfered with pitch identification at the earlier stages of categorization, a perceptual enhancement for falling tones in music was found for tonal speakers, similarly to what has been reported by the previous literature (e.g., Pfordresher and Brown, 2009; Giuliano et al., 2011; Bidelman et al., 2013).

In line with the model described by Xu et al. (2006) above, top–down modulation could account for the interference from linguistic categories on the perception of falling tones in language for speakers of Mandarin. Similar reasoning can be used to explain the perception of pitch contours in Dutch non-musicians. Non-musician speakers of Dutch tended to perform the worst of all three groups with no significant difference between accuracy rates in either domain. This group also demonstrated significantly less sharp boundaries of categorization for falling tones, and demonstrated no categorization effects for rising tones in either language or music. It was interesting to find that Dutch listeners demonstrated facilitation effects for falling tones in both speech and musical contexts, while showing the opposite pattern for rising tones.

That falling pitch contours appeared more salient to this group might be explained by the fact that intonational patterning and marking of phrase boundaries in the Dutch language shows considerable down-drift declination forcing sentential intonation downward as a sentence closes (e.g., Cohen and ’t Hart, 1967). Facilitation from sentential intonation might thus account for the higher degree of categorical identification. When we consider languages such as French, that typically show the opposite intonation pattern (i.e., up-drift) in intonation and phrase marking (Battye et al., 2000; cf. Patel, 2008), we might expect listeners to show a categorical enhancement for rising contours. Future research should investigate languages with different tonal inventories and intonation classifications in their categorical perception of rising and falling tonal continua.

Musicians demonstrated the highest accuracy rates, and sharp categorization of falling and rising tones in both speech and music. For musicians, pitch expertise appeared to extend from music to the language context with no significant differences between domains. Musicians generally appear to benefit from more fine-grained auditory acuity and sensory enhancement as a result of their musical training. This positive transfer of pitch acuity from the music to the language domain corresponds with earlier experimental findings that found experience with pitch in music to facilitate contour-tracking of lexical tones (Alexander et al., 2005; Lee and Hung, 2008; Delogu et al., 2010; Bidelman et al., 2011b; Marie et al., 2011; Mok and Zuo, 2012). It should be noted, however, that because we did not collect data on AP perception, nor control for other genetic predispositions (see Zatorre, 2013), conclusions on the role of musical expertise should be approached cautiously. While musicians out-performed both Mandarin speakers and non-musicians in terms of accuracy, *d*-prime sensitivity measures indicated both Mandarin speakers and Dutch musicians to demonstrate the highest sensitivity to pitch contour differences in both conditions.

Corroborating earlier findings by Wu et al. (2015), it is interesting to note that categorical boundary positions in speech and music appeared to differ significantly for non-musicians only. Given the more shallow categorization curves for rising tones in both contexts, it appears that categorical identification for non-musician speakers of Dutch is based more on bottom–up continuous sensory encoding, with a lesser degree of top–down modulation from intonational aspects of the language. Tonal perception for speakers of Mandarin on the other hand appears highly regulated by top–down expectations from lexical categories in language. Interestingly, categorical interference appears stronger for speakers of Mandarin (lexical effect) than for speakers of Dutch (intonational effect). Dutch musicians, on the other hand, appear able to balance between bottom–up matching, and top–down expectations, possibly as an effect of enhanced cognitive control or sensory acuity (Bialystok and DePape, 2009; Pallesen et al., 2010; Bidelman et al., 2013; Hutka et al., 2015).

It is striking that identification was far more categorical for the falling tone, opposed to the rising tone, between speech and music across all groups. It could be suggested that the frequent occurring arch-shaped contour of melody (i.e., a bell shaped tendency for melody to end on a falling contour; Huron, 1996) could have facilitated the perception of falling pitch contours. It would explain why we find a perceptual facilitation of this specific pitch contour in all groups, specifically in a musical context. This explanation offers certain merit as it fits perfectly in a model on top–down effects on categorical perception as suggested by Xu et al. (2006) above.

As a behavioral paradigm this study holds its limitations with regard to the interpretation of domain-specific perception. However, corroborating earlier findings, we have been able to demonstrate domain-specific top–down modulation of perceptual mechanisms to be operant when identifying pitch as either language or music (Abrams et al., 2011; Rogalsky et al., 2011; Merrill et al., 2012; Nan and Friederici, 2013; Tierney et al., 2013; Hutka et al., 2015). In light of the reported findings, it would be interesting to investigate the neural correlates and temporal dynamics that are operant when processing identical pitch patterns in either domain. Therefore, future studies using neuropsychological methods, such as EEG, should be conducted to further address the matter of domain-specific processing contrasting pitch perception in language and music.

## Conclusion

To our knowledge, this is the first study to address domain-specific perception of pitch in speech and music using identical pitch contours that are embedded in clearly demarcated linguistic and melodic phrases. Native speakers of a tone language such as Mandarin demonstrated clear domain-specific categorization patterns that are influenced by top–down lexical effects from language. Speakers of non-tonal languages such as Dutch did not show such clearly demarcated categorization, but appear influenced by intonational aspects of language. Musicians tend to treat pitch in both domains as equal. By priming listening mode, and directly contrasting between speech and music perception in speakers of tonal (Mandarin) and non-tonal (Dutch) languages, and in non-tonal speaking musicians, we provide support to a growing body of literature that suggest that experience with pitch in language and in music may exert significant influence on the manner in which pitch contours are categorized in either domain.

## Author Contributions

JW, MR-D, and HH designed the research; JW performed the research and analyzed the data; JW wrote the paper and MR-D and HH improved the paper.

## Conflict of Interest Statement

The authors declare that the research was conducted in the absence of any commercial or financial relationships that could be construed as a potential conflict of interest.
